# Comparison of the Effects of Environmental Parameters on the Growth Variability of *Vibrio parahaemolyticus* Coupled with Strain Sources and Genotypes Analyses

**DOI:** 10.3389/fmicb.2016.00994

**Published:** 2016-06-23

**Authors:** Bingxuan Liu, Haiquan Liu, Yingjie Pan, Jing Xie, Yong Zhao

**Affiliations:** ^1^College of Food Science and Technology, Shanghai Ocean UniversityShanghai, China; ^2^Laboratory of Quality and Safety Risk Assessment for Aquatic Products on Storage and Preservation, Ministry of AgricultureShanghai, China; ^3^Shanghai Engineering Research Center of Aquatic-Product Processing and PreservationShanghai, China

**Keywords:** *Vibrio parahemolyticus*, maximum growth rate, growth variability, environmental factor, temperature, salinity, gene heterogeneity

## Abstract

Microbial growth variability plays an important role on food safety risk assessment. In this study, the growth kinetic characteristics corresponding to maximum specific growth rate (μ_max_) of 50 *V. parahaemolyticus* isolates from different sources and genotypes were evaluated at different temperatures (10, 20, 30, and 37°C) and salinity (0.5, 3, 5, 7, and 9%) using the automated turbidimetric system Bioscreen C. The results demonstrated that strain growth variability increased as the growth conditions became more stressful both in terms of temperature and salinity. The coefficient of variation (CV) of μ_max_ for temperature was larger than that for salinity, indicating that the impact of temperature on strain growth variability was greater than that of salinity. The strains isolated from freshwater aquatic products had more conspicuous growth variations than those from seawater. Moreover, the strains with *tlh*^+^*/tdh*^+^*/trh*^−^ exhibited higher growth variability than *tlh*^+^*/tdh*^−^*/trh*^−^ or *tlh*^+^*/tdh*^−^*/trh*^+^, revealing that gene heterogeneity might have possible relations with the growth variability. This research illustrates that the growth environments, strain sources as well as genotypes have impacts on strain growth variability of *V. parahaemolyticus*, which can be helpful for incorporating strain variability in predictive microbiology and microbial risk assessment.

## Introduction

*Vibrio parahaemolyticus* is a kind of halophytic, Gram-negative bacterium that can cause headaches, diarrhea, fever, gastroenteritis, and even life-threatening sepsis (Makino et al., 2003). Since the first *V. parahaemolyticus* was isolated in Japan by Fujino Komiro in 1950 (Fujino et al., 1950), it has been considered as the major food-borne pathogen involving in bacterial seafood poisoning incidents in Asia (Fujikawa et al., 2009). According to annual statistics obtained from the detection network of microbial foodborne illness in China, *V. parahaemolyticus* has been classified as the major foodborne pathogen (accounting for 65% of the total; Wu et al., 2014). Similarly, cases of foodborne illness caused by *V. parahaemolyticus* are common in Europe and the United States (Yang et al., 2008; Shen et al., 2009, 2010). In fact, the largest outbreak of *V. parahaemolyticus* gastroenteritis all over the world did occur in the United States in 1978 and caused up to 1133 cases (Daniels et al., 2000). As demonstrated by recent surveillance data (Ma et al., 2014; Tang et al., 2014), the control of *V. parahaemolyticus* continues to be challenging worldwide.

It was announced that the strain variability gave the importance as well as the difficulty in controlling *V. parahaemolyticus* in the previous research (Lianou and Koutsoumanis, 2011). Owing to the fact that *V. parahaemolyticus* is mainly distributed in estuaries, coastal waters, sediments, and aquatic products (such as shrimp, cod, mackerel, and shellfish), it has become the major sources of food-borne pathogen (Wu et al., 2014). Since multiple strain composites of foodborne pathogens with robust growth or inactivation characteristics are preferred in food safety researches that aimed at assessing the behavior of bacterial pathogens in food products (NACMCF (National Advisory Committee on Microbiological Criteria for Foods), 2005; Scott et al., 2005), the characterizations of a variety of strains with respect to phenotypic responses, such as the growth behavior under different environmental conditions, should be analyzed (Nishina et al., 2004). Additionally, *V. parahaemolyticus* strains in the environment exhibit a halophilic and seasonal distribution, which are directly related to the salinity and temperature (DePaola et al., 2003; Zimmerman et al., 2007; Johnson et al., 2010; Sobrinho et al., 2014; Esteves et al., 2015). For the purpose of evaluation, the quantitative microbial risk assessment (QMRA) of *V. parahaemolyticus* should be estimated by at least two factors: temperature (T) and salinity (sodium chloride) (Nauta, 2002; U.S. Food and Drug Administration, 2005). It means that the use of predictive models of growth variability is mainly associated with the *T*-value and sodium chloride (NaCl) concentration (Ratkowsky et al., 1982; Larsen et al., 2015).

In previous studies of *V. parahaemolyticus*, only a few of them investigated the relationship between growth environments and strain variability (Fujikawa et al., 2009; Larsen et al., 2015). In west countries such as the USA, aquatic animals are mostly cultured in seawater (Depaola et al., 1990); however, in China, a majority of aquatic farmers practice freshwater aquaculture (Wu et al., 2014). It has been determined that the differences of the source for *V. parahaemolyticus* strains result in a large amount of diversity in the predictive models of growth variability (McMeekin et al., 1993). Furthermore, most previous research findings of the strain variability of the growth kinetic behavior of foodborne pathogens are based on marine culture (Wong et al., 2000; Alam et al., 2002; Larsen et al., 2015), which would be discrepant from China's actual conditions. Thus, new models with the purpose of developing a safe food production process in China should be built. Moreover, further studies on the influence of gene heterogeneity on growth variability were even less (Lianou and Koutsoumanis, 2013; Lopez-Joven et al., 2015), while as mentioned in Martins and Locke (2015), gene heterogeneity could determine phenotypic heterogeneity including strain growth variability, and therefore this variability might reflect the gene heterogeneity as well.

As the growth variability can introduce the food safety risk, the quantification of the growth variability can better service to the QMRA in microbiology. Aiming at furthering the development of precautionary food safety against *V. parahaemolyticus* in China, the influences of the T value and NaCl, together with strain sources and genotypes on the growth variability were evaluated in this research. The obtained appropriate data of the growth variability for *V. parahaemolyticus* could be useful for better characterizing the kinetic behaviors of *V. parahaemolyticus* in different growth environments (Miles et al., 1997; Yang et al., 2009). In total, 9000 optical density (OD) curves with 50 isolates of *V. parahaemolyticus* from different sources were generated for four levels temperatures and five levels NaCl concentrations, which will accomplish the following: (1) determine the influences of temperature and salinity on growth variability, and discuss the comparison between this two environmental factors; (2) reveal the growth variability of strains isolated from the aquatic products in freshwater and seawater; (3) demonstrate the effects of gene heterogeneity on the growth variability; and (4) provide a reasonable environmental condition for the storage of preserved food against *V. parahaemolyticus*.

## Materials and methods

### *V. parahaemolyticus* strains

Fifty strains of *V. parahaemolyticus* were isolated from the shrimps which were cultured in freshwater or seawater. The strain information was shown in Table 1. *tlh*^+^*/tdh*^+^*/trh*^−^, *tlh*^+^*/tdh*^−^*/trh*^+^, and *tlh*^+^*/tdh*^−^*/trh*^−^ genes were used for distinguishing the genotype of the isolates (Bej et al., 1999; Okada et al., 2009), twelve *V. parahaemolyticus* strains were *tlh*^+^*/tdh*^+^*/trh*^−^ genotype, eleven *V. parahaemolyticus* strains were *tlh*^+^*/tdh*^−^*/trh*^+^ genotype, one strain (42) was *tlh*^+^*/tdh*^+^*/trh*^+^ genotype and others were *tlh*^+^*/tdh*^−^*/trh*^−^ genotype in Table 1. All the strains in the present study were stored frozen (−80°C) in 25% glycerol test tubes. The *V. parahaemolyticus* strains were first dispensed onto thiosulfate-citrate-bile salts-sucrose agar culture medium (TCBS; Beijing Land Bridge Technology Company Ltd., Beijing, China) plates, and cultured for 18–24 h at 37°C. The single green strain on TCBS plates was then transferred into 10 ml tryptic soy broth (TSB; Beijing Land Bridge Technology Company Ltd., Beijing, China) with pH 8.0 and 3.0% (w/w) NaCl concentration. The 18 h cultures were incubated at 37°C for the preparation of the test inocula. The initial strain concentrations of the inocula were about 10^9^ CFU/ml after incubation. The automated turbidimetric system Bioscreen C (Oy Growth Curves Ab Ltd., Raisio, Finland) was used for testing the corresponding Optical density (OD) values. OD measurements were taken at regular time intervals using the wideband filter (420–580 nm) of the instrument, for a total time period such that a considerable OD change was observed.

**Table 1:** **The sources of the 50 strains of *V. parahaemolyticus* from the shrimps**.

**No**.	**Genotype**			**Source**	**No**.	**Genotype**			**Source**
	***tlh***	***tdh***	***trh***			***tlh***	***tdh***	***trh***	
1	+	−	+	Freshwater	26	+	−	−	Freshwater
2	+	−	−	Seawater	27	+	−	−	Freshwater
3	+	−	+	Freshwater	28	+	+	−	Freshwater
4	+	−	−	Freshwater	29	+	−	+	Freshwater
5	+	−	−	Seawater	30	+	−	+	Seawater
6	+	−	−	Seawater	31	+	+	−	Freshwater
7	+	+	−	Seawater	32	+	−	−	Seawater
8	+	−	+	Freshwater	33	+	−	−	Seawater
9	+	+	−	Seawater	34	+	−	−	Seawater
10	+	+	−	Seawater	35	+	−	+	Freshwater
11	+	−	−	Seawater	36	+	−	+	Freshwater
12	+	−	+	Freshwater	37	+	+	−	Seawater
13	+	+	−	Seawater	38	+	−	−	Freshwater
14	+	−	−	Seawater	39	+	−	−	Seawater
15	+	+	−	Seawater	40	+	−	+	Freshwater
16	+	−	−	Seawater	41	+	−	−	Freshwater
17	+	+	−	Seawater	42	+	+	+	Human
18	+	−	+	Freshwater	43	+	+	−	Human
19	+	−	−	Freshwater	44	+	−	−	Freshwater
20	+	−	+	Seawater	45	+	−	−	Seawater
21	+	−	−	Freshwater	46	+	−	−	Freshwater
22	+	−	−	Seawater	47	+	−	−	Freshwater
23	+	−	−	Freshwater	48	+	−	−	Freshwater
24	+	−	−	Seawater	49	+	−	−	Seawater
25	+	−	−	Seawater	50	+	−	−	Freshwater

*“+” represents positive genotypic, and “−” means negative genotypic*.

### Growth experiments

To evaluate the single effect of the T value or NaCl concentration on the growth variability in terms of the two environmental factors, a total of 20 different growth conditions were assessed with 4-levels (10, 20, 30, and 37°C) of temperature and 5-levels (0.5, 3, 5, 7, and 9%) of NaCl concentrations so as to cover the most probable growth region of the *V. parahaemolyticus* strains. The maximum and minimum boundaries of the T value (37 and 10°C, respectively) and the NaCl concentration (9 and 0.5%, respectively) were set up based on the findings of preliminary experiments in which the growth environment approximately reached the minimum growth requirements (*V. parahaemolyticus* strains approached the minimum growth rate in the condition of 10°C and 9% salinity) or the maximum growth requirements (*V. parahaemolyticus* strains attained the maximum growth rate in an optimum environment with 37°C and 3% salinity condition).The prepared initial inocula of each strain were decimally diluted in the TSB with 5 levels of NaCl concentration separately for five times. With strain concentration of approximately 10^4^ CFU/ml, the inoculated TSB were transferred into 100-well microtiter plates, which were then placed in the automated turbidimetric system Bioscreen C for 4 levels of temperatures, respectively. Totally three OD measurement replicates were tested in this process. Additionally, three independent experiments were conducted at each growth condition and therefore there were three samples per strain altogether for testing. In such a way, the total number of the described OD curves would amount to 9000 patterns (3 replicates × 3 independent experiments × 20 growth conditions × 50 types of *V. parahaemolyticus*). The counted data were analyzed in order to achieve an accurate approximation of the *V. parahaemolyticus* growth states in different cultured environments. Moreover, it would be more reasonable for the *V. parahaemolyticus* strain evaluation of QMRA (Vose, 1998).

### Maximum specific growth rate

The maximum specific growth rate (μ_max_) (Dalgaard and Koutsoumanis, 2001) of each strain at each growth condition was estimated according to Mytilinaios et al. (2012). By using the decimal dilution approach with Bioscreen C, the novel calculation of the maximum growth rate in the unit of OD^*^h^−1^ can be formulated in the model of Modified Gompertz (Gibson et al., 1987; Zwietering et al., 1990; Gil et al., 2006; Juneja et al., 2007; Yoon et al., 2008), with a little regulation, as the following equation:

$$\begin{array}{l} y=A+C\;exp\left\{-exp\left[\frac{\mu_{m}}{A}\left(\lambda -t\right)+1\right]\right\} \end{array}$$

Where *A* means the initial amount of bacteria, *C* represents the difference between the initial amount and the maximum amount of bacteria, μ_*m*_ represents maximum specific growth rate and λ is the lag time of the strain growth.

To calculate the maximum growth rate, the obtained data with both OD values and cultured times were taken into the above equation in the place of *y* and *t*, respectively. The OD curves were then fitted and the matrix of function was calculated including *A, C*, λ, and μ_*m*_.

### Statistical analysis

The statistical indicators were used to compare the performance of the models: correlation coefficients (*R*^2^), the p values from the Fisher *F*-test, and root mean square error (RMSE), accuracy factor (A_*f*_), and bias factor (B_*f*_), whose mathematical expressions are as follows:

$$\begin{array}{lll} R^{2} & = & \left[1-\frac{\sum {\left(pred-obs\right)}^{2}}{{\sum \left(obs-mean\right)}^{2}}\right]\\ RMSE & = & \sqrt{\frac{{\sum \left(obs-pred\right)}^{2}}{\;n}}\\ A_{f} & = & 10\left(\frac{\sum |Log\left(pred∕obs\right)|}{n}\right)\\ B_{f} & = & 10\left(\frac{\sum Log\left(pred∕obs\right)}{n}\right) \end{array}$$

where *obs* is observed values, *pred* is predicted values by models, *mean* is average values, and the *n* stands for the number of observations. The RMSE values approaching zero indicate a closer fit with the data for the model (Zhang et al., 2015). A_*f*_ provides the accuracy of the model, which reflects how close the predicted values are to the observed values, while B_*f*_ indicates the mean difference between observed and predicted value. Ideally, predictive models would have A_*f*_ = B_*f*_ = 1 (Wang et al., 2014).

The coefficient of variation (CV) of μ_max_ in different conditions were calculated within the formula as

$$\begin{array}{l} CV=\frac{{standard\;deviation\;of\;\mu }_{max}}{mean\;value\;of\;{\;\mu }_{max}}\;\times 100\% \end{array}$$

Significance testing making use of *p*-values was applied to verify the differences of the strain growth rate in different sources. Values differences were compared using the least significant difference (LSD) method at *p* = 0.05.Statistical analysis was performed using SPSS statistical package17.0 (SPSS Inc., Chicago, IL).

## Results

### Tendency of maximum growth rates

The estimated maximum specific growth rate μ_max_ vs. 50 strains in various growth environments were calculated are presented in Supplementary Material, and almost all of the values were fitted in the equation given above. By statistical analysis, all the correlation coefficients achieved above 93%, and all RMSE values approached zero. Both accuracy factors and bias factors got close to 1. The results showed a satisfactory goodness-of-fit in this study. A fraction of the maximal growth rate values could not yet be evaluated by Modified Gompertz model (Lammerding, 1997; Anderson and Hattis, 1999; Nauta and Dufrenne, 1999). It should be pointed out that the equation still cannot afford the actual growth state (Li et al., 2007), which requires the construction of a microbial macro growth model in multi-parameters.

**Figure 1:**
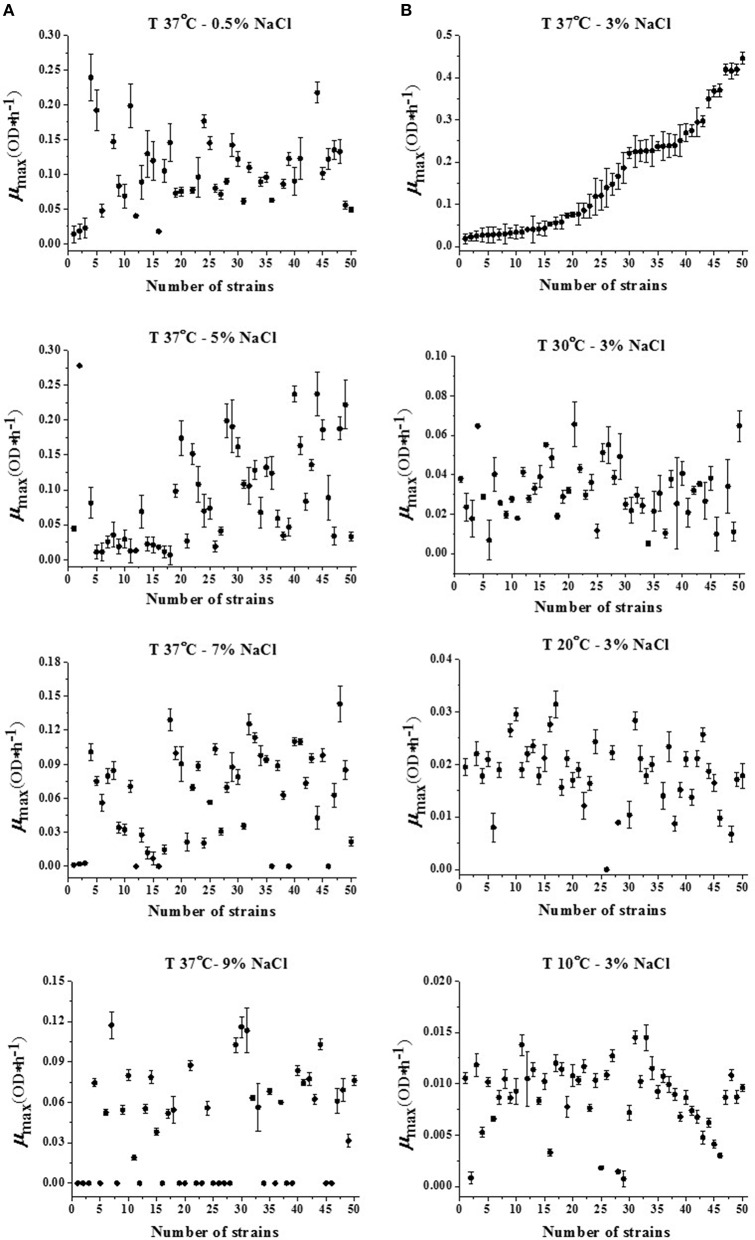
**Maximum specific growth rates (μ_max_) of 50 *V. parahaemolyticus* strains in different (A) NaCl concentrations (37°C) and (B) T values (3% NaCl)**.

Based on the μ_max_ in Supplementary Material, the tendency chats in various growth environments are shown in Figure 1. As shown in Figure 1A for the T of 37°C, the mean value of μ_max_ (OD^*^h^−1^) ranged from 0.03 to 0.24 in the condition of 0.5% NaCl, from 0.02 to 0.44 at 3% NaCl, from 0.01 to 0.26 at 5% NaCl, from 0 to 0.15 at 7% NaCl, and from 0 to 0.12 at 9% NaCl among the 50 strains. While with the same NaCl concentration of 3% in the TSB, the mean μ_max_ (OD^*^h^−1^) ranged from 0.02 to 0.44 at 37°C, from 0.005 to 0.065 at 30 °C, from 0.007 to 0.031 at 20°C, and from 0.001 to 0.014 at 10°C. Obviously, the average growth rate in the condition of 37°C and 3% NaCl concentration was found to be the largest (Figure 1B). Therefore, 37°C and 3% NaCl were considered as the optimal growth temperature and salinity respectively, similarly 10°C and 9% NaCl were considered as the most non-optimal temperature and salinity in this research.

### Evaluation of growth variability in different temperatures and salinities

The optimal growth condition at 37°C with 3% NaCl concentration was used as the reference in Figure 1. In this case, the strains from No. 1 to No. 50 tended to be staged growth with an increasing maximum specific growth rate μ_max_. While in other conditions, the strains from No. 1 to No. 50 seemed to grow randomly with no fixed growth trend as compared with that of the optimal growth condition. For example, the strains No. 50 and No. 1 at 37°C with 3% NaCl salinity had the highest growth rate and the lowest growth rate respectively, but in the condition at 20°C with 3% NaCl salinity, the No. 50 and No. 1 both located in the intermediate range of μ_max_ in all 50 strains, nearly 0.02 OD^*^h^−1^. Similar situations also appeared in other strains like No. 2, No. 13, No. 28 strains at 37°C with 5% NaCl salinity compared with those at 10°C with 3% NaCl concentration.

**Figure 2:**
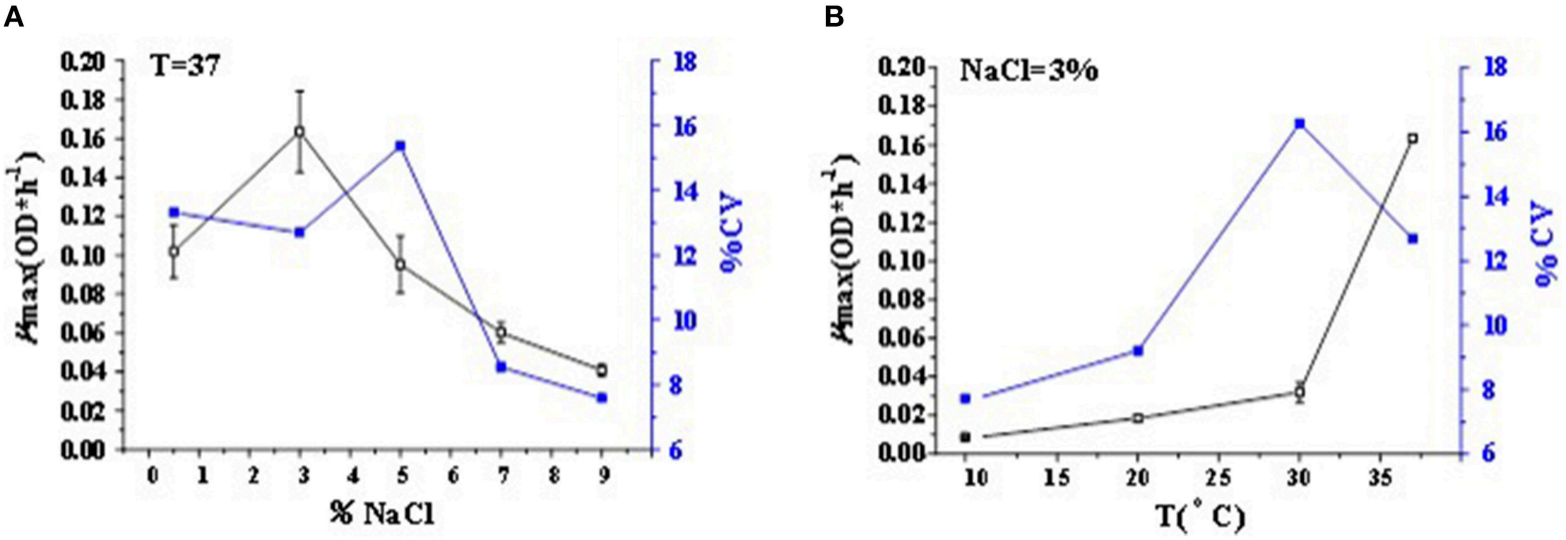
**Mean value curve of maximum specific growth rates (μ_max_) and coefficient of variation curve of μ_max_ among strains (CV-Strain) in different (A) NaCl concentrations and (B) *T*-values of *V. parahaemolyticus***.

The curves related to the coefficient of variation (CV) of μ_max_ in different conditions were drawn in Figure 2. The CV value of maximum growth rate represented the growth variability for *V. parahaemolyticus* strains. The CV value among the tested strains at 37°C-3% NaCl concentration was 12.7%, while at 37°C-0.5% NaCl concentration and T 37°C-5% NaCl concentration, it was 13.0 and 15.1%, respectively (Figure 2A). The CV value among the tested strains corresponding to a mean μ_max_ of approximately 0.16 OD^*^h^−1^ was 12.7% for 37°C-3% NaCl concentration, while corresponding to a mean μ_max_ of approximately 0.03 OD^*^h^−1^, the CV value was 16.3% for 30°C-3% NaCl concentration in Figure 2B. The non-optimal T and NaCl concentration led to an increase of CV values in the activation range of 0–5% NaCl and 30–37°C. On the contrary, in the inactivation range of *V. parahaemolyticus* strains, since the maximum growth rate dropped to nearly 0 OD^*^h^−1^, the CV values of μ_max_ would similarly drop down, with less variance of growth variability in *V. parahaemolyticus* strains as shown in the points among the 7–9% NaCl concentration and 10–20°C.

### Comparison of growth variability from different sources

**Figure 3:**
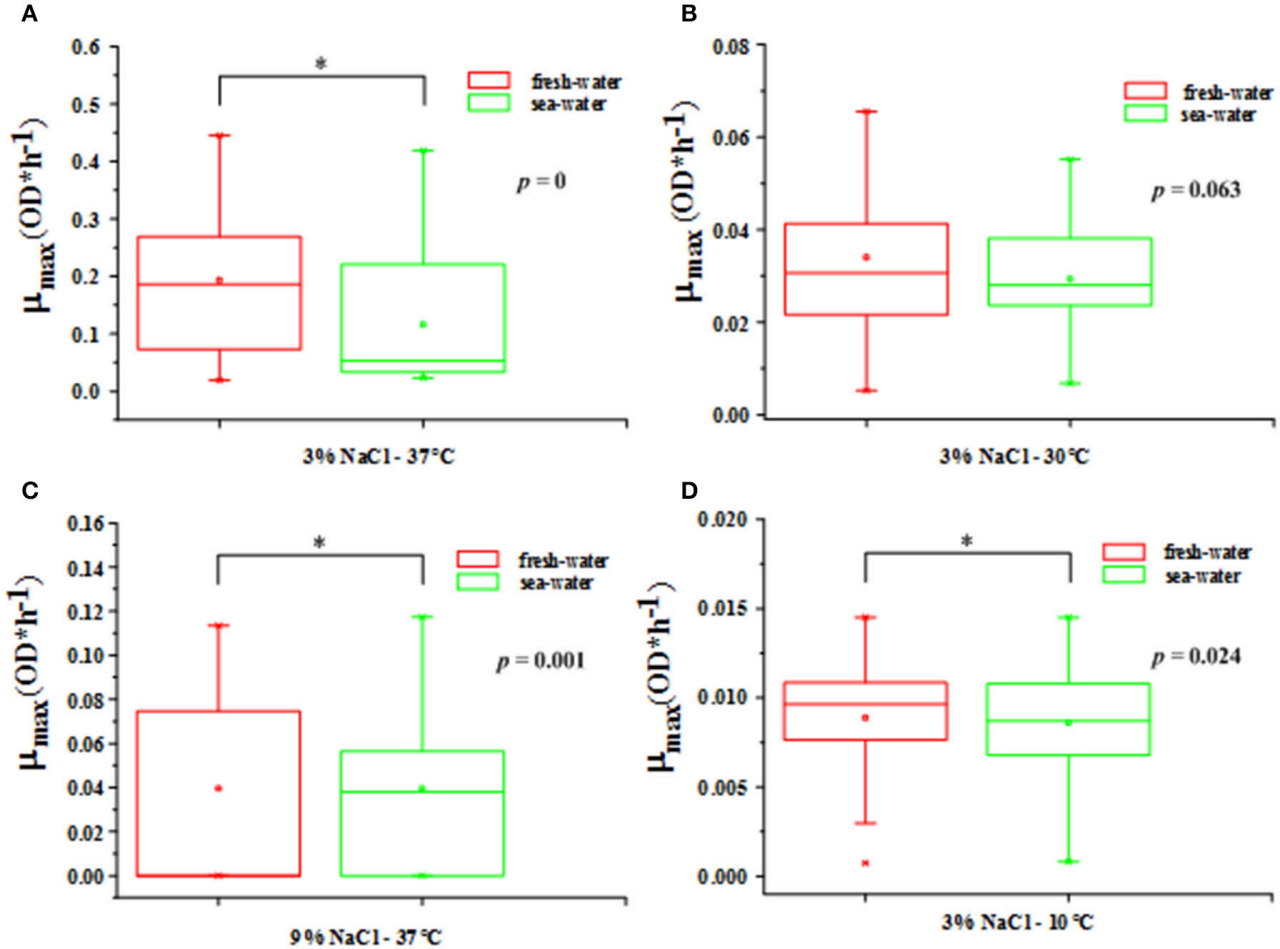
**The box plot between freshwater and seawater for *V. parahaemolyticus* strains in the shrimps in the following growth conditions. (A)** 37°C-3% NaCl concentration with *p* = 0 (optimal growth temperature and salinity), **(B)** 30°C-3% NaCl concentration with *p* = 0.063 (μ_max_ most consistent), **(C)** 37°C-9% NaCl concentration with *p* = 0.001 (optimal growth temperature and most non-optimal growth salinity), and **(D)** 10°C-3% NaCl concentration with *p* = 0.024 (most non-optimal growth temperature and optimal growth salinity). Statistical significance (*p* < 0.05) is shown by ^*^.

From the different types of environmental sources in Table 1, the *V. parahaemolyticus* strains could be roughly divided into two categories: freshwater and seawater. The significant difference analyses between these two categories in four environmental conditions (37°C-3% NaCl, 30°C-3% NaCl, 37°C-9% NaCl, and 10°C-3% NaCl) with in the box plot method were respectively drawn in Figure 3. The 4 environmental conditions represented 4 typical growth kinetics of *V. parahaemolyticus* strains. These four box plots were counted by the mean of the maximum growth rate μ_max_ in freshwater and seawater accordingly. In addition, the significant differences were calculated by *p*-value, with 0, 0.063, 0.001, and 0.024 respectively, which demonstrated the growth variability in these two sources. The non-significant difference occurred in the condition at 30°C-3% NaCl, and other conditions performed as the significant difference.

### Influence of genotypes on growth variability

**Figure 4:**
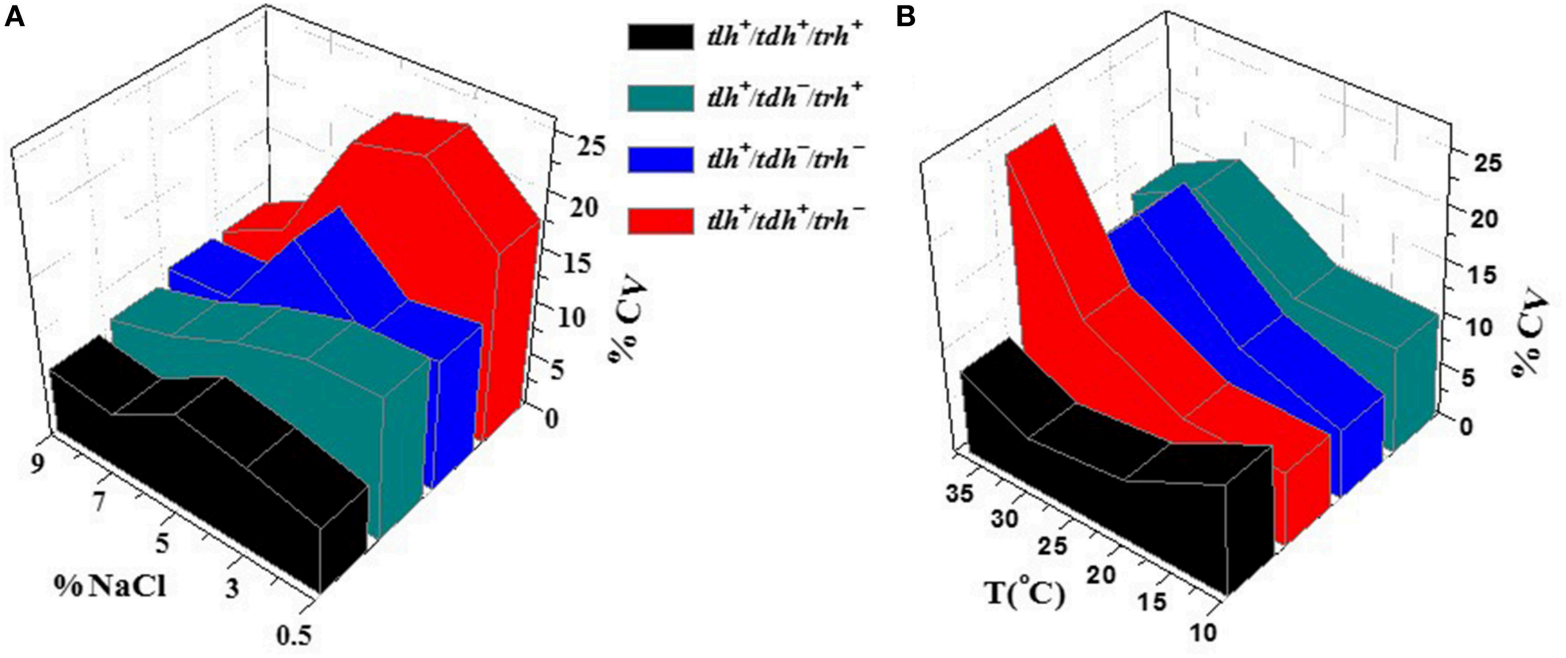
**The influence of the genotype on the growth variability of *V. parahaemolyticus* strains in various conditions. (A)** various NaCl concentrations with a fixed T value of 37°C and **(B)** various temperatures with a fixed NaCl concentration of 3%.

Further investigation of the growth variability of *V. parahaemolyticus* strains was studied through gene heterogeneity. For the purpose of research on the effect of genotypes on growth variability, all 50 strains were classified by growth condition and genotype. Based on each genotype with different virulence factors of *V. parahaemolyticus* strains (Letchumanan et al., 2014), four groups of virulence genes-related *V. parahaemolyticus* strains, *tlh*^+^*/tdh*^−^*/trh*^−^, *tlh*^+^/*tdh*^+^*/trh*^−^, *tlh*^+^/*tdh*^−^*/trh*^+^, and *tlh*^+^/*tdh*^+^*/trh*^+^, were introduced in this research in order to explore the internal causes of the growth variability of *V. parahaemolyticus*. Among these virulence genes, *tlh* has been expressed by all clinical and environmental strains of *V. parahaemolyticus* in previous studies (Bej et al., 1999; Okada et al., 2009); thus, the *tlh* virulence gene was contained in all four groups of isolates. The inter-specific variability of four genotype factors in the environmental factors of T and NaCl concentration is given in Figure 4. In Figure 4A, the temperature was fixed at the optimal condition of 37°C, and the genotype *tlh*^+^/*tdh*^+^*/trh*^−^ (colored in red) embodied the largest strain growth variability. The associated CV values were set at a high level compared with 3 other genotypes. In contrast, the *tlh*^+^/*tdh*^+^*/trh*^+^ genotype had the lowest CV values overall. Similar circumstances appeared in Figure 4B with the NaCl concentration set at 3% as well. The growth variability of *tlh*^+^*/tdh*^−^*/trh*^+^ (colored in green) and *tlh*^+^*/tdh*^−^*/trh*^−^ (colored in blue) performed moderate, overtopping the CV values only in the condition at 30°C and 3% NaCl concentration.

## Discussion

### Effects of temperature and NaCl concentration on μ_max_

It has been reported that the *V. parahaemolyticus* strains cannot grow at low temperature in nature since there is growth inhibition for *V. parahaemolyticus* strains with *T*-values below 10°C in freshwater or seawater (Cook and Ruple, 1989; Burnham et al., 2009). Regarding growth rate, the conditions at the lowest temperature (i.e., 10°C) and the highest NaCl concentration (i.e., 9%) almost approached the minimum growth requirements of this pathogen (Fujikawa et al., 2009). In contrast among all of the conditions, it is obvious that the growth condition of 37°C and 3% NaCl concentration is considered to be the optimal growth condition with the widest range and maximum mean value of specific growth rate compared to the other combinations (Figure 1), which has already been verified the similar completion in accordance with its own growth habits and laboratory experiments (Miles et al., 1997; Liu et al., 2008; Yang et al., 2009; Baker-Austin et al., 2010; Fernandez-Piquer et al., 2011). In this study, however, a much wider range of growth conditions in discussing both temperature and salinity simultaneously will give more comprehensive testimony for understanding the variability of the *V. parahaemolyticus* kinetic behavior within the scope of the growth environments.

It seemed that the inter-species growth variability of the 50 strains occurred at different environmental conditions. The trend of maximum growth rate in various conditions indicates that the external environment, such as the temperature and salinity, can affect the growth variability among *V. parahaemolyticus* strains, and such inter-species growth variability performs randomly (Whiting and Golden, 2002).

Moreover, because of incomplete knowledge of the effects of environmental conditions on model parameters in current microbiological studies (Nauta, 2002), the quantitative information based on a single impact parameter, like only the *T*-value or the NaCl concentration, needs to be evaluated separately in order to analyze the strain variability of *V. parahaemolyticus* (Fujikawa et al., 2009). Since there are no methods for separating two impact parameters absolutely, the mutual comparison between the one most non-optimal factor (*T*-value or NaCl concentration in this study) and the other factor in the optimal condition that emerged by the quantitative data is used here for approaching the actual microbial growth model (Lindqvist, 2006). As can be seen in the growth condition of 37°C-9% NaCl concentration, nearly half of the strains were inactive in such an inappropriate growth environment for *V. parahaemolyticus*, and it seemed that the rest of the strains still “struggled” in a random range of growth rate, mostly from 0.12 to 0, with a mean μ_max_ value of about 0.06; In another extreme condition with the most non-optimal temperature case while the optimal salinity: 10°C-3% NaCl concentration, although there were few inactive strains, the mean value of the growth rate μ_max_ could just achieve 0.01 or below, and μ_max_ ranged at a smaller scale from 0.015 to 0. The difference indicates that although the temperature and salinity have the same net effect on strain variability, meaning that the μ_max_ variability among the strains increases as the *T*-values or NaCl concentrations become more unfavorable for *V. parahaemolyticus* growth, the extent for this growth variability appears to be different for these two environmental parameters (Nauta, 2000). It is illustrated that as one of the two main impact parameters, the influence of low temperature on the decrease of μ_max_ appears to be greater than that of high NaCl concentration in Figure 1.

The analysis in Figure 1 could provide the formulations of temperature and salt which do not allow growth of *V. parahaemolyticus*. Normally, up to 9% NaCl concentration leads to inactivation for the majority of microorganisms (Francois et al., 2006); however, for halophilic bacteria like *V. parahaemolyticus*, such NaCl concentration cannot entirely prevent growth of bacteria (Anon, 1988; David et al., 1997). With a peak μ_max_ value of 0.12 for strain No. 7, 30, and 31, it seemed that the strain growth rate was not suppressed. To avoid the growth of most pathogens, the other impact parameter, the temperature, plays an important role in the suppression of μ_max_. As it was revealed above in the data from 20°C or even 10°C conditions, the *V. parahaemolyticus* strains in a low temperature could be more easily inactivated with weaker growth behavior. This suggests that preserved foods in a salty environment should be stored in low temperatures below 10°C, which can aid in avoiding *V. parahaemolyticus* growth. The present study gives a convincing data basis for the instruction of manufacturing preserved foods.

### Effects of temperature and NaCl concentration on growth variability

As reviewed by Nauta (2002), the assumption is often made by food microbiologists that strain-to-strain variation is equal to or smaller than experimental variation, thus it is not necessary to determine strain-to-strain variation. The data presented here demonstrated that the strain variability of the estimated μ_max_ values increased as the growth conditions became more stressful both in terms of NaCl (Figure 2A) and T (Figure 2B). The phenomenon that the non-optimal growth condition has a greater strain variability of growth kinetics than the optimal condition has been pointed in previous studies (Barbosa et al., 1994; Begot et al., 1997; Lianou et al., 2006).

In Figure 2 with both two cases, the maximum CV value occurred at the environmental condition of 30°C and 3% NaCl concentration. It meant that the growth variability of 50 strains was larger than that in the optimal growth condition and any other conditions. Thus, the condition might introduce much difficulties for the control of food safety risk. Actually, this condition comes closest to the natural environment, leading to a big challenge for food safety control (Pouillot et al., 2003). Nevertheless, the *V. parahaemolyticus* strains in this condition had a relative high consistency with the medium-to-high maximum growth rate, it might therefore maintain a large variety of serotypes as much as possible, which would achieve a diversity of *V. parahaemolyticus* strains with a similar growth rate when incubated in the same TSB. It exerted favorable effect on strain selection in the growth environment of 30°C and 3% NaCl concentration. Besides, with the similar maximum growth rate of 0.04 in the condition of T 37°C-9% NaCl in Figure 2A and the condition of T 30°C-3% NaCl in Figure 2B, the CV values in the two cases were quite different, corresponding to 3 and 16.27%, respectively, which meant that the growth variability was larger for decreasing temperature than for increasing salinity. It again proves the fact that temperature variation always leads to a more gradual increase in the growth variability in *V. parahaemolyticus* strains than NaCl variation.

### Impact of different sources on the growth variability

In Figure 3, the largest *p*-value for the difference between the freshwater and seawater occurred in the condition of 30°C and 3% NaCl, meaning this difference of μ_max_ was not significant. Obviously, this environment condition is the common state found in nature, especially in the subtropical and temperate coastal areas, which means that the *V. parahaemolyticus* strains that are grown in freshwater and in seawater result in a similar growth rate with relatively consistent growth variability in the normal state found in nature (Larsen et al., 2015). Moreover, the 30°C-3% NaCl condition could aid in strain selection since the consistency of the growth variability from fresh and sea water was optimal in all of the tested conditions, which offered the largest variety of growth variability. In other words, the natural environment found in the coastal areas will lead to a large growth variability in the *V. parahaemolyticus* strains, which results in difficulties for the QMRA and food safety control (Pouillot et al., 2003), as discussed in Figure 2.

Another interesting point is that there was an extremely significant difference in the growth variability of the freshwater and seawater *V. parahaemolyticus* strains in the environmental condition of 37°C and 3% NaCl concentration. It has been evaluated that the *V. parahaemolyticus* strains reached the largest maximum growth rate in such an optimal growth condition in Figure 3. On the contrary, the difference in the NaCl concentration caused the freshwater strains (living in 0.1% salinity in nature) having a larger growth variability than the seawater strains (living in 3% salinity in nature), which resulted in a higher mean value of maximum growth rate with a larger distribution according to an evaluation of its standard deviation. It has been stated that strains in a non-optimal environment condition will have growth variability at a considerably higher level than those in the optimal growth condition, and larger environmental pressure will lead to larger growth variability. In addition, based on the comparison between the condition of 37°C-9% NaCl concentration and that of 10°C-3% NaCl concentration, it is confirmed that a decreasing temperature leads to a somewhat more gradual decrease of μ_max_ than an increasing NaCl concentration does.

### Influence of different genotypes on the growth variability

According to collected data from Table 1, since there was only one strain comprising *tlh*^+^*/tdh*^+^*/trh*^+^, it should have no typical representativeness for the properties of this genotype, while the curve in red gave some reference for the tendency of different genotypes in Figure 4. From the results, it was concluded that the genotype of the *tlh*^+^/*tdh*^+^*/trh*^−^ resulted in the largest variation degree in the growth variability of the *V. parahaemolyticus* strains in all four groups, whereas the genotype with *tlh*^+^/*tdh*^+^*/trh*^+^ illustrated the least obvious variation degree from among those cultured in the environment condition with temperature and NaCl concentration, which verified that gene heterogeneity also affected the growth inter-specific variability for *V. parahaemolyticus*. Furthermore, as one of the major virulence genotypes in *V. parahaemolyticus, tlh*^+^/*tdh*^+^*/trh*^−^ modeled the most non-optimal case in evaluating QMRA, due to there being a large risk of growth variability in reality. In addition, it is suggested that in the food safety control of clinical *V. parahaemolyticus* strains, more attention should be paid to the genotype of *tlh*^+^/*tdh*^+^*/trh*^−^, which is associated with serious virulence (Miyamoto et al., 1969; Honda and Iida, 1993; Baffone et al., 2005) and large growth variability in most of the environmental conditions. The research exploring the effect of gene heterogeneity on the grow variability in *V. parahaemolyticus* provides a useful reference for the prevention of pathogenic *V. parahaemolyticus* in nature.

## Conclusion

In the present study, the growth kinetics characteristics of 50 *V. parahaemolyticus* isolates with different sources and genotypes were assessed at different temperatures (10, 20, 30, and 37°C) and salinity (0.5, 3, 7, and 9%). From the experimental results, it was concluded that the strain variability increased as the growth conditions became more stressful both in terms of temperature and salinity in the activation range, and temperature has larger impacts than salinity on strain growth variability. Therefore, the preserved foods in a salty environment were suggested to be stored in a low temperature below 10°C, which could promise the inactivation of *V. parahaemolyticus* strains. Moreover, the results showed the fact that the strains isolated from freshwater aquatic product had more conspicuous variations than those from seawater. And it was interpreted that gene heterogeneity also affected strain growth variability of *V. parahaemolyticus*. The findings of this study should be useful in incorporating strain variability in predictive microbiology and microbial risk assessment, and could provide scientific guidance for *V. parahaemolyticus* verification and prevention in nature as well as strain selection in experiments.

## Author contributions

BL performed the data analyses and wrote the manuscript; contributed significantly to analysis and manuscript preparation; HL helped perform the analysis with constructive discussions; Substantial contributions to the design of the work and analysis the results. YP, JX drafted the work or revising it critically for important intellectual content. Agreement to be accountable for all aspects of the work in ensuring that questions related to the accuracy or integrity of any part of the work are appropriately investigated and resolved. YZ contributed to the conception of the study. Drafting the work or revising it critically for important intellectual content. Final approval of the version to be published.

## Funding

This research was supported by the National Natural Science Foundation of China (31271870, 31571917), the project of Science and Technology Commission of Shanghai Municipality (14DZ1205100, 14320502100), Key Project of Shanghai Agriculture Prosperity through Science and Technology (2014, 3-5 and 2015, 4-8), Shanghai Engineering Research Center of Aquatic-Product Processing & Preservation (11DZ2280300), and the “Dawn” Program of Shanghai Education Commission (15SG48).

### Conflict of interest statement

The authors declare that the research was conducted in the absence of any commercial or financial relationships that could be construed as a potential conflict of interest.
