# Supplementary material for: Comparison of the Effects of Environmental Parameters on the Growth Variability of Vibrio parahaemolyticus Coupled with Strain Sources and Genotypes Analyses
Source: Front Microbiol. 2016 Jun 23;7:994. doi: 10.3389/fmicb.2016.00994 (PMC4917555; doi:10.3389/fmicb.2016.00994)
Supplement: Supplementary file 1 [file Table1.PDF]

# Supplementary Material

**Table 1:** The maximum growth rate values ($\mu_{max}$) of 50 strains at different NaCl concentrations and different T values:

a) T = 10 ℃

| Strain | % NaCl (T = 10 ℃ ) | | | | | Strain | % NaCl (T = 10 ℃ ) | | | | |
|---|---|---|---|---|---|---|---|---|---|---|---|
| | **0.5** | **3** | **5** | **7** | **9** | | **0.5** | **3** | **5** | **7** | **9** |
| No.1 | 0.00228 | 0.01056 | 0.00833 | 0.01447 | 0.00019 | No.26 | 0.00598 | 0.01086 | 0.00427 | 0.00866 | 0.00028 |
| No.2 | 0.00242 | 0.00082 | - | 0.00319 | 0.00029 | No.27 | - | 0.01273 | 0.00333 | 0.01528 | 0.00012 |
| No.3 | - | 0.01184 | 0.00762 | 0.00383 | 0.00016 | No.28 | 0.00904 | 0.00145 | 0.00407 | 0.00000 | 0.00038 |
| No.4 | 0.00073 | 0.00527 | 0.00075 | 0.02294 | 0.00032 | No.29 | - | 0.00073 | 0.00623 | 0.00298 | 0.00019 |
| No.5 | 0.01134 | 0.01018 | 0.00464 | 0.00823 | 0.00009 | No.30 | 0.00000 | 0.00719 | 0.00315 | 0.00456 | 0.00029 |
| No.6 | 0.00685 | 0.00659 | 0.00317 | 0.00000 | 0.00028 | No.31 | 0.01050 | 0.01451 | 0.00858 | 0.00297 | 0.00016 |
| No.7 | 0.01060 | 0.00867 | 0.00342 | 0.01388 | 0.00012 | No.32 | 0.00419 | 0.01023 | 0.00125 | 0.00000 | 0.00032 |
| No.8 | 0.00340 | 0.01046 | 0.00747 | 0.00500 | 0.00038 | No.33 | 0.00126 | 0.01451 | 0.00128 | 0.00172 | 0.00009 |
| No.9 | 0.01249 | 0.00862 | 0.00454 | 0.01338 | 0.00000 | No.34 | 0.00000 | 0.01151 | 0.00473 | 0.00027 | 0.00028 |
| No.10 | 0.00394 | 0.00927 | 0.00589 | 0.00000 | 0.00021 | No.35 | 0.01588 | 0.00923 | 0.00311 | 0.00264 | 0.00012 |
| No.11 | 0.00000 | 0.01379 | 0.00722 | 0.02048 | 0.00019 | No.36 | 0.00334 | 0.01074 | 0.00716 | 0.02789 | 0.00038 |
| No.12 | - | 0.01050 | 0.00623 | 0.00867 | 0.00029 | No.37 | 0.00759 | 0.00993 | 0.00184 | 0.02571 | 0.00019 |
| No.13 | 0.00027 | 0.01138 | 0.00560 | 0.00000 | 0.00016 | No.38 | 0.00000 | 0.00896 | 0.00457 | 0.00268 | 0.00029 |
| No.14 | 0.00746 | 0.00834 | 0.00405 | 0.00000 | 0.00032 | No.39 | 0.00051 | 0.00678 | 0.00325 | 0.00000 | 0.00016 |
| No.15 | 0.01052 | 0.01025 | 0.00910 | 0.00000 | 0.00009 | No.40 | 0.00313 | 0.00867 | 0.00672 | 0.00901 | 0.00032 |
| No.16 | 0.00485 | 0.00330 | 0.00574 | 0.00936 | 0.00028 | No.41 | 0.00348 | 0.00738 | 0.00191 | 0.01447 | 0.00009 |
| No.17 | 0.00907 | 0.01202 | 0.00399 | 0.00958 | 0.00012 | No.42 | - | 0.00676 | 0.00275 | 0.00220 | 0.00028 |
| No.18 | - | 0.01143 | 0.00247 | 0.00043 | 0.00038 | No.43 | 0.00056 | 0.00475 | 0.00335 | 0.01438 | 0.00019 |
| No.19 | 0.00009 | 0.00775 | 0.00419 | 0.01231 | 0.00000 | No.44 | 0.00069 | 0.00622 | 0.00280 | 0.03283 | 0.00029 |
| No.20 | 0.00486 | 0.01078 | 0.00638 | 0.00026 | 0.00000 | No.45 | 0.00000 | 0.00411 | 0.00335 | 0.00000 | 0.00016 |
| No.21 | 0.00003 | 0.01035 | 0.00579 | 0.00000 | 0.00019 | No.46 | 0.00056 | 0.00297 | 0.00446 | 0.00000 | 0.00032 |
| No.22 | 0.00000 | 0.01170 | 0.00221 | 0.00000 | 0.00029 | No.47 | 0.00355 | 0.00867 | 0.00142 | 0.00000 | 0.00009 |
| No.23 | 0.00399 | 0.00763 | 0.00488 | 0.00397 | 0.00016 | No.48 | 0.00099 | 0.01084 | 0.00424 | 0.00000 | 0.00028 |
| No.24 | 0.01384 | 0.01036 | 0.00304 | 0.00000 | 0.00032 | No.49 | 0.00000 | 0.00870 | 0.00431 | 0.00000 | 0.00012 |
| No.25 | 0.00000 | 0.00179 | 0.00355 | 0.00960 | 0.00009 | No.50 | 0.00374 | 0.00963 | 0.00325 | 0.00000 | 0.00038 |

- No fitting curve in the model of Modified Gompertz with the exact $\mu_{max}$ value.

b) T = 20 ℃

| **Strain** | **% NaCl (T = 20 ℃ )** | | | | | **Strain** | **% NaCl (T = 20 ℃ )** | | | | |
|---|---|---|---|---|---|---|---|---|---|---|---|
| | **0.5** | **3** | **5** | **7** | **9** | | **0.5** | **3** | **5** | **7** | **9** |
| No.1 | 0.03200 | 0.01950 | 0.01400 | 0.01800 | 0.01500 | No.26 | 0.03395 | 0.00000 | 0.01215 | 0.01725 | 0.01389 |
| No.2 | 0.03750 | 0.02000 | 0.02371 | - | - | No.27 | 0.02772 | 0.02223 | 0.01518 | 0.01651 | 0.00489 |
| No.3 | 0.04100 | 0.02200 | 0.02400 | 0.02180 | 0.01200 | No.28 | 0.00895 | 0.00895 | 0.00800 | 0.01929 | 0.00943 |
| No.4 | 0.03198 | 0.01778 | 0.01485 | 0.01578 | 0.00260 | No.29 | 0.02879 | 0.01230 | 0.00692 | - | - |
| No.5 | 0.01200 | 0.02097 | 0.01586 | 0.01923 | 0.00110 | No.30 | 0.02346 | 0.01036 | 0.00501 | 0.00885 | 0.00725 |
| No.6 | 0.03900 | 0.00800 | 0.00600 | 0.01200 | - | No.31 | 0.03234 | 0.02834 | 0.00961 | 0.02057 | -- |
| No.7 | 0.03301 | 0.01895 | 0.01184 | 0.02066 | 0.00796 | No.32 | 0.03589 | 0.02109 | 0.01271 | 0.01824 | 0.00843 |
| No.8 | 0.03090 | 0.02714 | 0.01021 | 0.00965 | 0.00000 | No.33 | 0.02241 | 0.01781 | 0.01388 | 0.01689 | 0.00806 |
| No.9 | 0.03536 | 0.02646 | 0.01881 | 0.01901 | 0.01533 | No.34 | 0.03900 | 0.02000 | 0.02000 | 0.01600 | 0.01900 |
| No.10 | 0.03684 | 0.02952 | 0.02145 | 0.01860 | 0.01638 | No.35 | 0.03541 | 0.02796 | 0.01921 | 0.01492 | - |
| No.11 | 0.03600 | 0.01900 | 0.01800 | 0.01800 | 0.01400 | No.36 | 0.03500 | 0.01400 | 0.01800 | 0.02100 | 0.01500 |
| No.12 | 0.04190 | 0.02200 | 0.01700 | 0.01900 | 0.01600 | No.37 | 0.03212 | 0.02338 | 0.01696 | 0.01921 | 0.01846 |
| No.13 | 0.02646 | 0.02351 | 0.01757 | 0.01623 | 0.01486 | No.38 | 0.02607 | 0.00872 | 0.00547 | 0.01322 | 0.00709 |
| No.14 | 0.02880 | 0.01779 | 0.01313 | 0.01629 | 0.00559 | No.39 | 0.03151 | 0.01515 | 0.01087 | 0.01495 | 0.00253 |
| No.15 | 0.03210 | 0.02120 | 0.01071 | 0.01865 | 0.00654 | No.40 | 0.03200 | 0.02100 | 0.01400 | 0.02100 | 0.01700 |
| No.16 | 0.03033 | 0.02760 | 0.01771 | 0.01553 | 0.00252 | No.41 | 0.02852 | 0.01372 | 0.00986 | 0.02362 | 0.00530 |
| No.17 | 0.02647 | 0.03143 | 0.02437 | 0.01675 | 0.01360 | No.42 | 0.03264 | 0.02112 | 0.01695 | 0.01937 | 0.00918 |
| No.18 | 0.01637 | 0.01560 | 0.00895 | 0.01787 | 0.00906 | No.43 | 0.03190 | 0.02567 | 0.01568 | 0.01692 | - |
| No.19 | 0.03053 | 0.02110 | 0.02867 | 0.01933 | 0.00933 | No.44 | 0.03583 | 0.01869 | 0.01008 | 0.01570 | - |
| No.20 | 0.03700 | 0.01700 | 0.02100 | 0.02160 | 0.01400 | No.45 | 0.02664 | 0.01646 | 0.00420 | 0.01334 | 0.01314 |
| No.21 | 0.03400 | 0.01900 | 0.01500 | 0.01800 | 0.01400 | No.46 | 0.02250 | 0.00977 | 0.00981 | 0.01660 | 0.01409 |
| No.22 | 0.00895 | 0.01212 | 0.00564 | 0.01770 | 0.01000 | No.47 | 0.02139 | 0.01543 | 0.00872 | - | - |
| No.23 | 0.03012 | 0.01637 | 0.03134 | 0.02243 | 0.01271 | No.48 | 0.02333 | 0.00671 | 0.01091 | 0.01392 | 0.00777 |
| No.24 | 0.03666 | 0.02429 | 0.01238 | 0.01010 | 0.01757 | No.49 | 0.01785 | 0.01713 | 0.01125 | 0.01683 | 0.01142 |
| No.25 | 0.03579 | 0.02110 | 0.01221 | 0.00824 | 0.00643 | No.50 | 0.02677 | 0.01781 | 0.01345 | 0.01388 | - |

- No fitting curve in the model of Modified Gompertz with the exact $\mu_{max}$ value.

c) T = 30 ℃

| Strain | % NaCl (T = 30 ℃) | | | | | Strain | % NaCl (T = 30 ℃) | | | | |
|---|---|---|---|---|---|---|---|---|---|---|---|
| | **0.5** | **3** | **5** | **7** | **9** | | **0.5** | **3** | **5** | **7** | **9** |
| No.1 | 0.02581 | 0.03792 | 0.03234 | 0.01740 | 0.01622 | No.26 | 0.05614 | 0.05135 | 0.02848 | 0.00731 | 0.02109 |
| No.2 | 0.05783 | 0.02359 | 0.01522 | 0.02199 | 0.01710 | No.27 | 0.05356 | 0.05510 | 0.03117 | 0.01788 | 0.02208 |
| No.3 | 0.04166 | 0.01771 | 0.02176 | 0.03802 | 0.01779 | No.28 | 0.04383 | 0.03861 | 0.01195 | 0.02046 | 0.02201 |
| No.4 | 0.05833 | 0.06465 | 0.03867 | 0.01442 | 0.01854 | No.29 | 0.02884 | 0.04919 | 0.02994 | 0.02179 | 0.01728 |
| No.5 | 0.05129 | 0.02890 | 0.02033 | 0.01793 | 0.01520 | No.30 | 0.03340 | 0.02514 | 0.02084 | 0.01896 | 0.02129 |
| No.6 | 0.06000 | 0.00680 | 0.00340 | 0.00221 | 0.01039 | No.31 | 0.04505 | 0.02182 | 0.02042 | 0.01766 | 0.02277 |
| No.7 | 0.04187 | 0.04033 | 0.01892 | 0.02862 | 0.01914 | No.32 | 0.01692 | 0.02963 | 0.03011 | 0.01138 | 0.02394 |
| No.8 | 0.04800 | 0.02580 | 0.01740 | 0.01380 | 0.00890 | No.33 | 0.04485 | 0.02436 | 0.01688 | 0.01366 | 0.01858 |
| No.9 | 0.01520 | 0.01980 | 0.01360 | 0.01220 | 0.00390 | No.34 | 0.01222 | 0.00519 | 0.01367 | 0.02652 | 0.01888 |
| No.10 | 0.01980 | 0.02780 | 0.01550 | 0.01010 | 0.00083 | No.35 | 0.05500 | 0.02159 | 0.02351 | 0.02309 | 0.02195 |
| No.11 | 0.01560 | 0.01800 | 0.01380 | 0.00190 | 0.00620 | No.36 | 0.04368 | 0.03069 | 0.02475 | 0.02237 | 0.02253 |
| No.12 | 0.02762 | 0.04132 | 0.02880 | 0.02472 | 0.01750 | No.37 | 0.01958 | 0.01039 | 0.04199 | 0.01451 | 0.02001 |
| No.13 | 0.01900 | 0.02800 | 0.01900 | 0.00390 | 0.00210 | No.38 | 0.04945 | 0.03769 | 0.02473 | 0.01700 | 0.01976 |
| No.14 | 0.02200 | 0.03300 | 0.01700 | 0.01400 | 0.01200 | No.39 | 0.03969 | 0.02542 | 0.02400 | 0.00296 | 0.01941 |
| No.15 | 0.04810 | 0.03896 | 0.02245 | 0.02117 | 0.01970 | No.40 | 0.03547 | 0.04068 | 0.03286 | 0.01993 | 0.01782 |
| No.16 | 0.03843 | 0.05522 | 0.03839 | 0.02174 | 0.01637 | No.41 | 0.03534 | 0.02083 | 0.02876 | 0.01067 | 0.01038 |
| No.17 | 0.02978 | 0.04855 | 0.03266 | 0.02098 | - | No.42 | 0.07723 | 0.03206 | 0.03125 | 0.00344 | 0.02364 |
| No.18 | 0.01205 | 0.01900 | 0.02342 | 0.01566 | 0.00727 | No.43 | 0.02570 | 0.03538 | 0.03748 | 0.00267 | 0.02388 |
| No.19 | 0.01389 | 0.02890 | 0.00935 | 0.01251 | 0.01914 | No.44 | 0.05935 | 0.02667 | 0.02981 | 0.00188 | 0.01461 |
| No.20 | 0.02114 | 0.03204 | 0.02187 | 0.03328 | 0.01747 | No.45 | 0.06007 | 0.03819 | 0.01721 | 0.01772 | 0.01455 |
| No.21 | 0.04600 | 0.06560 | 0.03044 | 0.02427 | 0.01874 | No.46 | 0.03541 | 0.01000 | 0.01921 | 0.01242 | 0.01899 |
| No.22 | 0.02982 | 0.04319 | 0.03768 | 0.01001 | - | No.47 | 0.04498 | 0.01973 | 0.03230 | 0.01603 | 0.02268 |
| No.23 | 0.03309 | 0.02975 | 0.01159 | 0.00384 | - | No.48 | 0.05093 | 0.03417 | 0.01172 | 0.00910 | 0.02439 |
| No.24 | 0.02014 | 0.03616 | 0.02944 | 0.02136 | 0.01741 | No.49 | 0.04031 | 0.01105 | 0.01418 | 0.00321 | - |
| No.25 | 0.04250 | 0.01166 | 0.00145 | 0.01325 | 0.02242 | No.50 | 0.04897 | 0.06477 | 0.02793 | 0.01804 | 0.01769 |

- No fitting curve in the model of Modified Gompertz with the exact $\mu_{max}$ value.

d) T = 37 ℃

| Strain | % NaCl (T = 37 ℃ ) | | | | | Strain | % NaCl (T = 37 ℃ ) | | | | |
|---|---|---|---|---|---|---|---|---|---|---|---|
| | 0.5 | 3 | 5 | 7 | 9 | | 0.5 | 3 | 5 | 7 | 9 |
| No.1 | 0.01380 | 0.01887 | 0.04464 | 0.00120 | 0.00000 | No.26 | 0.07997 | 0.13951 | 0.01867 | 0.10347 | 0.00000 |
| No.2 | 0.01820 | 0.02312 | 0.27800 | 0.00230 | 0.00000 | No.27 | 0.07133 | 0.14737 | 0.04106 | 0.03085 | 0.00000 |
| No.3 | 0.02290 | 0.02477 | 0.32200 | 0.00280 | 0.00000 | No.28 | 0.08954 | 0.16654 | 0.19912 | 0.06971 | 0.00000 |
| No.4 | 0.23933 | 0.02695 | 0.08109 | 0.10097 | 0.07447 | No.29 | 0.14197 | 0.18626 | 0.19078 | 0.08756 | 0.10281 |
| No.5 | 0.19204 | 0.02755 | 0.01086 | 0.07514 | 0.00000 | No.30 | 0.12201 | 0.22118 | 0.16153 | 0.07882 | 0.11595 |
| No.6 | 0.04753 | 0.02798 | 0.01100 | 0.05600 | 0.05257 | No.31 | 0.06151 | 0.22432 | 0.10816 | 0.03580 | 0.11345 |
| No.7 | 0.07290 | 0.02855 | 0.02574 | 0.07968 | 0.11731 | No.32 | 0.10972 | 0.22573 | 0.10561 | 0.12553 | 0.06340 |
| No.8 | 0.14691 | 0.02976 | 0.03550 | 0.08457 | 0.00000 | No.33 | - | 0.22615 | 0.12823 | 0.11349 | 0.05650 |
| No.9 | 0.08333 | 0.03252 | 0.01876 | 0.03423 | 0.05428 | No.34 | 0.08919 | 0.22658 | 0.06762 | 0.09789 | 0.00000 |
| No.10 | 0.06865 | 0.03367 | 0.02935 | 0.03240 | 0.08012 | No.35 | 0.09555 | 0.23724 | 0.13244 | 0.09429 | 0.06825 |
| No.11 | 0.19875 | 0.03384 | 0.01203 | 0.07035 | 0.01898 | No.36 | 0.06279 | 0.23777 | 0.12379 | 0.00000 | 0.00000 |
| No.12 | 0.04018 | 0.04018 | 0.01300 | 0.00000 | 0.00000 | No.37 | - | 0.23851 | 0.05911 | 0.08873 | 0.05998 |
| No.13 | 0.08911 | 0.04033 | 0.06891 | 0.02772 | 0.05532 | No.38 | 0.08611 | 0.23959 | 0.03431 | 0.06302 | 0.00000 |
| No.14 | 0.12963 | 0.04146 | 0.02245 | 0.01220 | 0.07870 | No.39 | 0.12249 | 0.25113 | 0.04655 | 0.00000 | 0.00000 |
| No.15 | 0.11958 | 0.04289 | 0.02120 | 0.00703 | 0.03810 | No.40 | 0.09000 | 0.26892 | 0.23719 | 0.11028 | 0.08355 |
| No.16 | 0.01780 | 0.05304 | 0.01780 | 0.00000 | 0.00000 | No.41 | 0.12257 | 0.27485 | 0.16374 | 0.10973 | 0.07460 |
| No.17 | 0.10483 | 0.05628 | 0.01105 | 0.01465 | 0.05189 | No.42 | - | 0.29421 | 0.08349 | 0.07339 | 0.07737 |
| No.18 | 0.14575 | 0.05826 | 0.00655 | 0.12937 | 0.05443 | No.43 | 0.20970 | 0.29689 | 0.13595 | 0.09532 | 0.06253 |
| No.19 | 0.07305 | 0.07305 | 0.09832 | 0.09975 | 0.00000 | No.44 | 0.21746 | 0.34969 | 0.23776 | 0.04278 | 0.10295 |
| No.20 | 0.07560 | 0.07560 | 0.17398 | 0.09053 | 0.00000 | No.45 | 0.10161 | 0.36772 | 0.18628 | 0.09792 | 0.00000 |
| No.21 | - | 0.07687 | 0.02682 | 0.02136 | 0.08759 | No.46 | 0.12201 | 0.36991 | 0.08873 | 0.00000 | 0.00000 |
| No.22 | 0.07714 | 0.08536 | 0.15148 | 0.06950 | 0.00000 | No.47 | 0.13496 | 0.41893 | 0.03390 | 0.06282 | 0.06088 |
| No.23 | 0.09611 | 0.09611 | 0.10793 | 0.08850 | 0.00000 | No.48 | 0.13235 | 0.41598 | 0.18778 | 0.14341 | 0.06921 |
| No.24 | 0.17653 | 0.11834 | 0.06973 | 0.02053 | 0.05592 | No.49 | 0.05583 | 0.41922 | 0.22215 | 0.08503 | 0.03154 |
| No.25 | 0.14505 | 0.12060 | 0.07342 | 0.05653 | 0.00000 | No.50 | 0.04942 | 0.44553 | 0.03301 | 0.02177 | 0.02780 |

- No fitting curve in the model of Modified Gompertz with the exact $\mu_{max}$ value.
